# Peri-partum psychosis: How does parental reflective function affect the quality of mother-infant interaction?

**DOI:** 10.4102/sajpsychiatry.v24i0.1302

**Published:** 2018-09-20

**Authors:** Juané Voges, Astrid Berg, Daniel J.H. Niehaus

**Affiliations:** 1 Department of Psychiatry, Faculty of Medicine and Health Sciences, Stellenbosch University, South Africa; 2 Stikland Hospital, South Africa; 3 Department of Psychiatry and Mental Health, Faculty of Health Sciences, University of Cape Town, South Africa

## Abstract

**Introduction:**

The purpose of this exploratory study was to examine the impact of peri-partum psychosis on parental reflective function and quality of mother-infant interaction in a South African sample at high risk of developing attachment difficulties. Besides the effects of physical separation, attachment difficulties may arise from other maternal factors, such as a lack of reflective capacity or negative symptoms affecting the warmth with which a mother interacts with her child. This study examined the quality of mother-infant interaction to determine how the presence of psychotic symptoms during pregnancy or shortly after delivery affects aspects like maternal sensitivity, child social involvement and dyadic engagement. Ultimately, the study aimed to investigate the association between psychosis, parental reflective functioning and quality of parent-infant interaction.

**Methods:**

The study followed a descriptive, observational design. Mothers were recruited if they experienced psychotic symptoms during pregnancy or within the first 6 months postpartum. Demographic information and psychiatric history were collected. Parental reflective function was assessed by the Parent Development Interview (PDI), and the quality of mother-infant interaction in an unstructured play interaction was coded using the Coding Interactive Behaviour (CIB).

**Results:**

Eight participants aged between 22 and 44 years, with diagnoses of bipolar disorder, schizophrenia and MDD with psychosis, were recruited. Parental reflective functioning showed significant variation with scores approaching and exceeding ordinary reflective functioning, typically found in non-clinical populations. At the time of the play interaction, infants were aged between 6 and 10 months. Play interactions were mostly parent-led, with some surprising findings, such as a lower than anticipated frequency of parental negative affect, moderate maternal sensitivity and wide variation in scores for infant withdrawal.

**Conclusion:**

The results found in this small sample of mothers with peri-partum psychosis have presented unexpected results, both in terms of higher than anticipated capacity for parental reflective functioning and aspects of the quality of interaction with their infants. Possible implications for future interventions will be discussed.

